# “No research about us, without us:” a qualitative study to engage with community research advisory boards in the urban community

**DOI:** 10.3389/fpubh.2026.1825508

**Published:** 2026-06-22

**Authors:** Chakema Carmack, Damien Kelly, Kristen Morrison, Arlette Chavez, Ashika Brinkley, Pablo Panta

**Affiliations:** 1 Health Center for Addictions Research and Cancer Prevention, University of Houston, Houston, TX, United States; 2 Psychological Health and Learning Sciences Department, University of Houston, Houston, TX, United States

**Keywords:** community engagement, community research advisory board, health disparities, advocacy, urban

## Abstract

**Background:**

Community Advisory Boards (CABs) and Community Research Advisory Boards (CRABs) are widely used in health research to promote both ethical conduct and build community trust. Despite their growing adoption by universities, limited empirical work has examined how these advisory boards experience engagement with academic researchers.

**Purpose:**

This qualitative study explored best practices for engaging CRABs serving urban communities, drawing on the experiences of members of the University of Houston HEALTH Research Center's community research advisory board.

**Methods:**

Seven CRAB members participated in two virtual focus groups conducted between November 2024 and February 2025. Participants brought professional expertise across public health, social work, healthcare, and community advocacy. Data were transcribed verbatim and analyzed using reflexive thematic analysis to identify connecting patterns in participants' experiences, expectations, and perceptions of the academic/community research relationship.

**Results:**

Four themes emerged from the research: (1) Urban Communities Want Feedback and Sustained Engagement, (2) Urban Communities Feeling Ignored or Used by Researchers, (3) Urban Communities Want Advocacy and Resources, not Just an Identification of the Problem, and (4) Consequences and Benefits for the Community Connector.

**Conclusions:**

Findings highlight that meaningful engagement with urban communities requires reciprocal, sustained partnerships that extend beyond data collection. CRABs can serve as trusted conduits between universities and urban communities when researchers intentionally incorporate community feedback. Additionally, closing communication loops and translating research into actionable benefits can increase community engagement and show that dissemination of academic findings is a priority.

## Introduction

Community research advisory boards (CRAB) and community advisory boards (CAB) are increasingly recognized as vital components in research, serving as a safeguard against exploitation, shaping the conduct of studies, and enhancing dissemination efforts (1–4). These advisory boards play a crucial role in ensuring that research is ethically sound and aligned with the needs of the communities involved. Beyond their protective function, CRABs and CABs are instrumental in the practical implementation of research, particularly in areas such as participant recruitment and serving as a liaison between researchers and community members (1). By engaging directly with these community stakeholders, researchers provide a platform for community members to voice their perspectives, fostering greater transparency and mutual understanding between the research team and the communities they aim to serve.

### Community advisory boards (CABs) and community research advisory boards (CRABs)

CABs and CRABs have become critical components in health research, particularly in ensuring that research practices align with community needs and ethical standards. CABs may include neighborhood organizations, faith-based organizations, health centers, clinicians, clinic patients, and others. The role of these advisory boards is multifaceted, encompassing responsibilities such as safeguarding against exploitation, influencing study design, and enhancing the relevance and dissemination of research findings. Despite their increasing adoption in academic and health research fields, the effectiveness of CABs and CRABs varies significantly, largely due to challenges related to their structure, training, and integration within the research process.

CABs have been used traditionally in practice to assist researchers and research projects in addressing community needs in their efforts to positively affect communities. However, CABs are increasingly being utilized across multiple projects, broadening their scope and reach in community research. Wilczek et al. (5) conducted a scoping review of research review boards (RRBs), which operate similarly to CABs, though across the scope of many projects and are meant to complement institutional review boards. Of the 25 studies that were included in the review analysis, the authors found several similarities and differences in the approaches and evaluations used within RRBs. At their core, all RRBs were unified in their mission toward the protection of the community when participating in research. They were consistently designed to support researchers across various stages of project development and execution within the community. The majority of the boards were formed in university settings, and a smaller number were community-based. While boards formed in university settings were typically funded through university and federal funds, community-based RRBs cited issues of sustainability, as their funding structure typically included private or foundational funding. University-formed boards faced challenges related to academic leadership and to conflicts of interest among board members. Evaluation and feedback were noted as areas of divergence across the RRBs, with only a few reporting these criteria. Among those who provided evaluation data, researchers reported satisfaction with working with RRBs and intentions to continue their partnerships. However, assessing the impact of the RRBs remains challenging due to the lack of systematic evaluation within individual boards as well as across RRBs. Moving beyond community protection to community benefit should be the priority of CABs and RRBs, ensuring that communities reap the benefits of the research and are properly educated regarding the research findings and their impact on the community (5).

Stewart et al. (4) surveyed 44 federally funded health science center programs utilizing CABs on seven research-developed domains: membership and selection (e.g., the types of members and how they are identified), training and documentation (e.g., meeting agendas and operating principles), roles and responsibilities in advisement, CAB influence and operations (e.g., frequency of meetings, length, service terms), feedback and input (e.g., to what extent CAB feedback is incorporated), evaluation of the CAB, and perceived benefits and challenges. They found that 86% were recruited mainly through referrals from other CAB members and 75% through word-of-mouth. Seventy-five percent of the CABs were provided with information on their roles and responsibilities. Common activities included recruitment for research studies, raising awareness, and dissemination of the research to communities. Similar to Wilczek et al. (5), this study found that many CABs reported that they maintained influence over their own operations, such as member selection, setting agendas, and documentation. CAB meetings were held either quarterly, monthly, or bi-monthly for an average of 1–2 h. Seventy-seven percent of the CABs compensated their CAB members, with varying payment amounts and payment structures. The majority of the CTSAs reported that they evaluated their CABs on attendance, the frequency of meetings, and the quality of the recommendations received, but satisfaction surveys and interviews of the CAB members were implemented less frequently. Regarding challenges faced by CAB partnerships, CABs reported that competing priorities limited their participation in scheduled meetings, and some reported uncertainty about how to meaningfully contribute to the CAB. Communications between meetings and feeling undervalued were cited as challenges to sustained buy-in and engagement (4).

Brockman et al. (1) provided insight into how CABs impact various stages of the research process, including study design, implementation, and dissemination. Their study highlights that while CABs may significantly influence study design and implementation, their impact on other research stages, such as analysis, is less pronounced. There is an emphasis on the need for enhanced engagement strategies to maximize the influence of CABs across all research stages (1, 5). This finding is echoed by Ortega et al. (3), who explored the motivations and challenges faced by CAB members in a community-academic partnership in Washington State. Ortega et al. found that while CAB members are motivated by a commitment to improving community health, their challenges include inconsistent communication and unclear roles that limit their effectiveness. The study underscored the importance of clear roles and consistent engagement to sustain the effectiveness of CABs. Relatedly, Matthews et al. (6) conducted a study on the community engagement advisory board (CEAB), similar to CABs and CRABs, at the University of Illinois at Chicago. In their findings, the researchers highlighted areas for improvement, such as the need for better onboarding and training for new members. The CEAB members also expressed a desire for feedback on how their contributions were used by researchers.

Though many reviews conclude that more research is needed on standardizing the structure and operations of CABs, fewer examine the perceptions of the CAB regarding their participation in CABs. In a large, federally funded study that utilized CAB participation, researchers sought to understand how participation affected *N* = 19 CAB members in various public health domains participating in a large opioid reduction study (7). The study found that the CAB members viewed the CAB meetings as an open and honest space to discuss community concerns as they related to the research. CAB members looked forward to attending the regularly scheduled meetings. The knowledge gained from participating in CAB was mutually beneficial to both the CAB and the academic researchers. Harm reduction, as a working model of the CAB's purpose, unified CAB members in how to better serve their communities. CAB members reported being grateful for the knowledge they received from other CAB members and appreciated broadening their perspectives regarding the research topics. Their participation in the CAB also allowed for networking opportunities outside of the research that would assist in furthering the research's efforts and impact on the community (e.g., reducing opioid addiction). The regularity of the meetings (once per month over two years) facilitated regular communication and inspired connectedness and belonging toward a community health improvement mission.

These studies provided a foundation for how CABs and CRABs operate in academic institutions, their responsibilities and structure, and common challenges experienced among them. Overall, the most-reported contributions of the CAB included building partnerships between academia and the community, building trust between universities and the community, advising on community health priorities, representing community interests, and increasing community engagement. However, fewer reported helping the community understand research, increasing research participation of underrepresented populations, and disseminating back into the community (4, 5).

### About the University of Houston HEALTH community research advisory board

The Community Engagement Core at the University of Houston's HEALTH Center for Addictions Research and Cancer Prevention (HEALTH), an NIH-designated Research Center in Minority Institutions (RCMI), includes provisions for a CRAB that meets bi-monthly (twice per month) in order to assist the center's researchers with the implementation of research projects, recruitment, dissemination, relevance, and other factors concerning community health. The CRAB members provide essential feedback to affiliated investigators, guiding academic health research projects from their inception and design through to the dissemination of results. The mission of the HEALTH CRAB is stated as: *The CRAB works to enhance research projects (all stages) and proposals to ensure their feasibility within the community and assist researchers to translate their work back into the community in a timely manner*. The CRAB is composed of a diverse group of community members who professionally serve in many community health sectors, such as addiction recovery advocates, cancer survivors, community caregivers, and individuals passionate about addressing the unique health challenges that disproportionately impact community members. CRAB members participate as individuals, rather than representatives of their affiliated organizations or employers. Members of the CRAB receive training from the Community Engagement Core, ensuring they are well-versed in research ethics, design, and implementation.

### Purpose of the study

Despite the growing adoption of CABs and CRABs in health research, there is a noticeable lack of studies that examine the most effective strategies for engaging with these boards. This paper aims to address this gap by presenting a qualitative study focused on the University of Houston's HEALTH Center's engagement with its CRAB and the broader communities they represent. The study aims to explore best practices for engaging with CRABs and CABs participating in research in urban communities. We seek to contribute to understanding best practices and offer insights into how CRABs can be leveraged to strengthen the research processes and outcomes and meaningfully contribute to community health within urban communities.

## Methods

### Participants

The CRAB consisted of ten board members (9 women and 1 man). Of the ten board members, *N* = 7 females participated in the study. Although participants' ages ranged was not captured, participants' length of experience in community advocacy ranged from 10 to 30 years of experience. The first focus group (or dyadic interview) (*N* = 1) lasted approximately one hour, while the second focus group (*N* = 6) lasted approximately 1 h and 30 min. Their backgrounds included public health, community engagement, social work, and healthcare. Many members were involved in leadership roles within their organizations, such as directors of health initiatives, non-profit leaders, and educators. Their work spanned areas including substance abuse prevention, cancer research, health disparities, mental health, and community advocacy. This diverse mix of fields enhanced the board's ability to address complex health issues within the community effectively.

### Measures

The study utilized a researcher-developed interview instrument with 12 open-ended questions. The questions posed were carefully formulated based on a thorough review of existing literature relevant to the subject. By grounding the questions in previously published research, we aimed to ensure they not only address key issues identified by experts in the field but also contribute to the ongoing scholarly discourse. This approach allows the study to build upon established knowledge while exploring new dimensions of the topic. The questions and citations are listed below in Table 1.

**Table 1:** Citations and questions.

Citation	Research question
Israel et al. (13); Williams and Mohammed (14)	What are the primary health concerns and unique challenges faced by the urban community in your area?
Minkler et al. (15); Macaulay et al. (16)	How do you perceive the relationship between UH researchers and the community and what recommendations do you have for improving this partnership?
Cargo and Mercer (17)	What factors do you believe contribute to successful community engagement in research?
Corbie-Smith et al. (18)	What strategies do you believe are effective in building trust between researchers and the urban community?
Green and Mercer (19); Jagosh et al. (2)	How do you feel about the way research findings are communicated back to the community and what role do you think community members should play in the design and implementation of research studies?
Flicker and Worthington (20)	What ethical considerations do you think are important in community-based research?
Wallerstein and Duran (21)	How do you think research can better address the needs and priorities of your community?
Viswanathan et al. (22)	What barriers do you see to effective collaboration between researchers and the community?
Goodman and Sanders Thompson (23)	How do you define success in a community-based participatory research project?
Minkler et al. (15)	How do you perceive the impact of research on improving health outcomes in your community?
Brownson et al. (24); Schulz et al. (25)	How do you think research findings should be used to inform policy decisions and how do you feel about the long-term sustainability of research initiatives in your community?
Israel et al. (26)	What are the main challenges your community may face in participating in research activities and how can they be addressed?

### Data collection and data analysis

CRAB participants were previously recruited and approved to sit on the advisory board for the Community Engagement Core of the HEALTH research center for a 2-year term. These participants were selected for this study using purposive sampling based on their membership on the CRAB. Participants were approached for participation via email and informed that the purpose of the study was to explore best practices for strengthening community-research partnerships and CRAB engagement within urban communities. Two online focus groups were recorded and transcribed via Zoom from November 2024 to February 2025. One participant attended the November 2024 focus group, while six participants attended the February 2025 focus group. The same seven members did not attend both sessions.

All CRAB participants who participated in the study signed an informed consent form upon agreeing to be in the study. Focus group sessions were audio recorded, and Chakema Carmack, Ph.D., moderated the focus group sessions. Dr. Carmack has prior experience in community-based participatory research, qualitative interviewing/focus group moderation, and reflexive thematic analysis. The audio recordings were transcribed verbatim. No qualitative data analysis software was used; coding and thematic analysis were conducted manually by the research team. The research team consisted of four females and two males, all with academic experience in working with CABs and CRABs.

First, all research team members (i.e., the authors) engaged in open reflexive thematic coding of the data independently. The authors then convened in two separate meetings to discuss their developed themes and reflections. Upon group agreement, statements were categorized into themes and subthemes with supporting dialogue. Following preliminary theme development, findings were presented to back to CRAB members during a follow-up meeting in September 2025 to obtain feedback, clarify interpretations, and identify any needed corrections or additions. All CRAB members were in agreement with the themes developed and were grateful for the opportunity to reflect upon the process and connections that were made during the interpretation of the data.

Reflexive thematic analysis (RTA), developed by Braun and Clarke (8), is a method used by researchers to develop and report themes within the data. It allowed the researchers to interpret the data while acknowledging their backgrounds as a source of reflection in data interpretations. The benefit of RTA is its flexibility, whereas other analysis methods, such as grounded theory, are theoretically bound. However, RTA can be adapted to various theoretical frameworks and research questions. Because themes are developed and do not “emerge,” RTA does not lend itself to traditional thematic analysis metrics such as saturation or rater agreement indices. The present study utilized the COREQ (COnsolidated criteria for REporting Qualitative research) Checklist (9) to ensure that the study properly captured the research team and reflexivity (Domain 1), appropriate study design information (Domain 2), and analysis and findings (Domain 3).

## Results

Themes were inductively derived from participant narratives through reflexive thematic analysis. Through this analysis, four themes were developed: Urban Communities Want Feedback and Sustained Engagement; Urban Communities Feeling Ignored or Used by Researchers; Urban Communities Want Advocacy and Resources, not Just an Identification of the Problem; and Consequences and Benefits for the Community Connector. The following themes are organized with reflexive analyses and direct quotes (DQ) supporting the descriptions.

### Theme 1: urban communities want feedback and sustained engagement

According to researchers Ellis and Muyita (10), community connectors are a trusted person from within the community who helps build relationships between researchers and community members. Our community connectors spoke of wanting more feedback from researchers and being informed of their research facilitation from beginning to end. Some spoke of empty promises of advocacy and showing up, but then getting what they want (e.g., participant data) and leaving without a trace. Lack of consistency in the reporting back to the community was an issue that was repeated throughout the discourse. Community connectors rightfully complained about “minimal involvement” in the creation of the research and researchers “not truly being of help.” Sustained engagement, historical mistrust, lack of understanding of community constituents, and lack of consistent feedback were commonly mentioned throughout. There is an importance for the community to be involved in the whole research facilitation timeline, including the dissemination of the results. Participants noted that good engagement is happening, and they want to see more of it: more UH representation, collaborations, and support for the benefit of the community. Overall, participants expressed the importance of keeping the community informed to build trust and accountability.

DQ (Direct Quote)1: *The community wants UH to share research outcomes and progress with the community. Ways we can share with the community: meetings, events, forums, health fairs, etc. would like to see more of that with reference to various studies*.

DQ2: *It perpetuates the mistrust if you come in do a research project and then don't follow up with any type of solution or answer to the problem. So just reminding the researchers that that's an important part of the whole, you know, continuum to make sure that they‘re thinking about POST Research. What is the response to the problems that are found within the research that they're doing?*

DQ3: …*But then the verbiage changes once they get the information that is needed, everything changes. Those communities are no longer part of that research, or if they are, it's very minimal*.

### Theme 2: urban communities feeling ignored or used by researchers

A heavy focus on recruitment numbers and not “the people” was very important to the participants. They spoke about the community feeling ignored or used by researchers when they are left in the dark about research studies. A fond and exciting relationship is established, it runs its course for several weeks, and months later, the community can't remember the researcher's name. Participants spoke about how this formula did not build “genuine” relationships. Notwithstanding, engaging the community before you need them for research is key. The community yearns to be involved in the primary steps of research that is done on them, supposedly for them. Communities feel unheard and undervalued. “Ignored” showed up many times in the transcriptions of dialogue. Communities experience frustration stemming from the lack of inclusion, specifically when the exclusion is perceived to be due to where they are, geographically.

Participants perceived a lack of cultural humility and a lack of understanding from researchers, particularly about The Historic Third Ward in the Houston community, to which the University of Houston community research serves, and the surrounding communities' concerns.

DQ1: *You can't go into the community and say, “This is what you need.” No, you have to say “tell me what it is that you need,” and that's how I would recommend improving it that*

DQ2: …*But we are focused on targeted zip codes. The zip codes that actually need it, the ones that don't want to go into those, are the ones I'm going into [like] Sunnyside and The Third Ward. Well, we are always in the Third Ward, and that's a great thing. The Fourth Ward, that is on the other side of [highway] 288. We are missing those zip codes*.

DQ3: *I think it's important that they [*urban *community members] have a voice in research regarding the community. The health concerns in the community are the lack of consistency and the biases that go along with that*.

### Theme 3: urban communities want advocacy and resources, not just an identification of the problem

Urban communities face many socio-environmental health issues that researchers are aware of. Participants discussed how university researchers are doing research, but not advocating for these community issues. There are resources that people are physically close to but can't access (e.g., healthy food stores, free low-cost clinics). The community expressed a need to know how to access these resources, and in some ways, will look to the researchers claiming to help them for such information. The research alone doesn't improve access on its own. Participants spoke about the value of being committed to all of it, including the public health and community improvement aspect of the research, and not just sample size goals for research. They spoke of wanting researchers to acknowledge systematic geographic health service gaps. Pooling research, or even discussing with the community, tangible resources, and the potential for policy reform, whether on the local community or state level, is needed from researchers requesting individual-level data from community members. Researchers should send leaders information about resources and access. Meeting people at their own level came up adjacent to this theme in many instances. Community connectors and researchers are trying to evoke a sense of caring in the community. Asking them what *they* need is crucial as well.

DQ1: *You can build this trust and rapport, but you have to include them in the beginning*.

DQ2: *I think it's important that they have a voice in ‘what is research' regarding the community. The health concerns are a lot of everything*.

DQ3: *If they [*urban *community members] were at the table, they would show researchers how to make it more relatable for them [*urban *community members]. Some of our community members have a high school education, some dropped out, even young people that we're trying to get this info. We're trying to do research and provide equity straight across the board, [but] we have to know our audience.”*

DQ4: *Why do so many communities say they're stuck? Because the majority of the people are on government assistance. When they get their groceries, they are buying their food all at one time. If a hurricane comes in the middle of the month, they don't have the money. They don't have the resources to go to HEB [grocery store] and buy another case of water or whatever they may need to sustain them*.

### Theme 4: consequences and benefits for the community connector

There were mixed results on the perceived benefits of the community connector. Some community leaders who served as community connectors for our center's university research expressed positive sentiments regarding their experience with the CEC. Particularly, that our CEC maintains good relationship building, aside from the community wanting more study-related briefings and interactions. As a couple of participants noted:

DQ1: *I have received so much in the way that you all cultivate relationships and maintain them is a two-way street, which is obviously very important. If the council would need something, you know, we would go all-out for you all; or if y'all need something, and you come out to us. And it's not just, I mean, with the HEALTH CRAB, yes, but the whole university too*.

DQ2: *I love what UH [University of Houston] stands for: going into the community and really doing something. I absolutely love it, but I would love to see more consistency*.

However, not all community connectors shared this sentiment. Some spoke of the relationship between researchers and the community connectors as exploitive, mentioning that, when you exploit the relationship, it has an effect on their relationships. Some participants spoke about poor connections with our CEC and a desire to improve the relationship. It is important to take care of that relationship because it has a domino effect. This may be detrimental and fracture the relationship, resulting in less trust. In such circumstances, the CEC may be less likely to have a reliable and effective community partner. Long-term, it benefits no one.

DQ3: *I actually have the opposite experience. Just to be honest, I think our relationship, or my relationship with UH, is very different from the relationship with UT [University of Texas] School of Public Health and Rice University. I feel that maybe it's the placement or location of where my health centers are [located] relative to the proximity of UH campus. Maybe we‘re not the right target population sometimes that UH is looking for, but I feel that we're not included in a round table discussion of how we can work on meaningful projects together. So, I have the opposite experience, and with the other universities, I'm actually sitting around table discussions, talking about opportunities and how we can impact the community together*.

DQ4: *You get the information that you want, and then you tell them, “We‘re going to take this information, and we're going to put it toward research, and well, thank you very much.” Now the group of people that I have a relationship with will look to me and say, “Okay, so what are they going to do next?”*

It is important for us to think deeply about these relationships with the community and our community leaders and connectors. The community connector can play a role by engaging the research with the community, but researchers need to engage with the CRAB so that they can be a more effective conduit with the community. As one participant articulated:

DQ5: *So, I stand in the middle. I stand in the middle making sure that I'm hearing what's going on in research, and then I'm taking that in the community and making sure, like telling them and informing them, hey, look, this is what's going on*.

**Figure 1:**
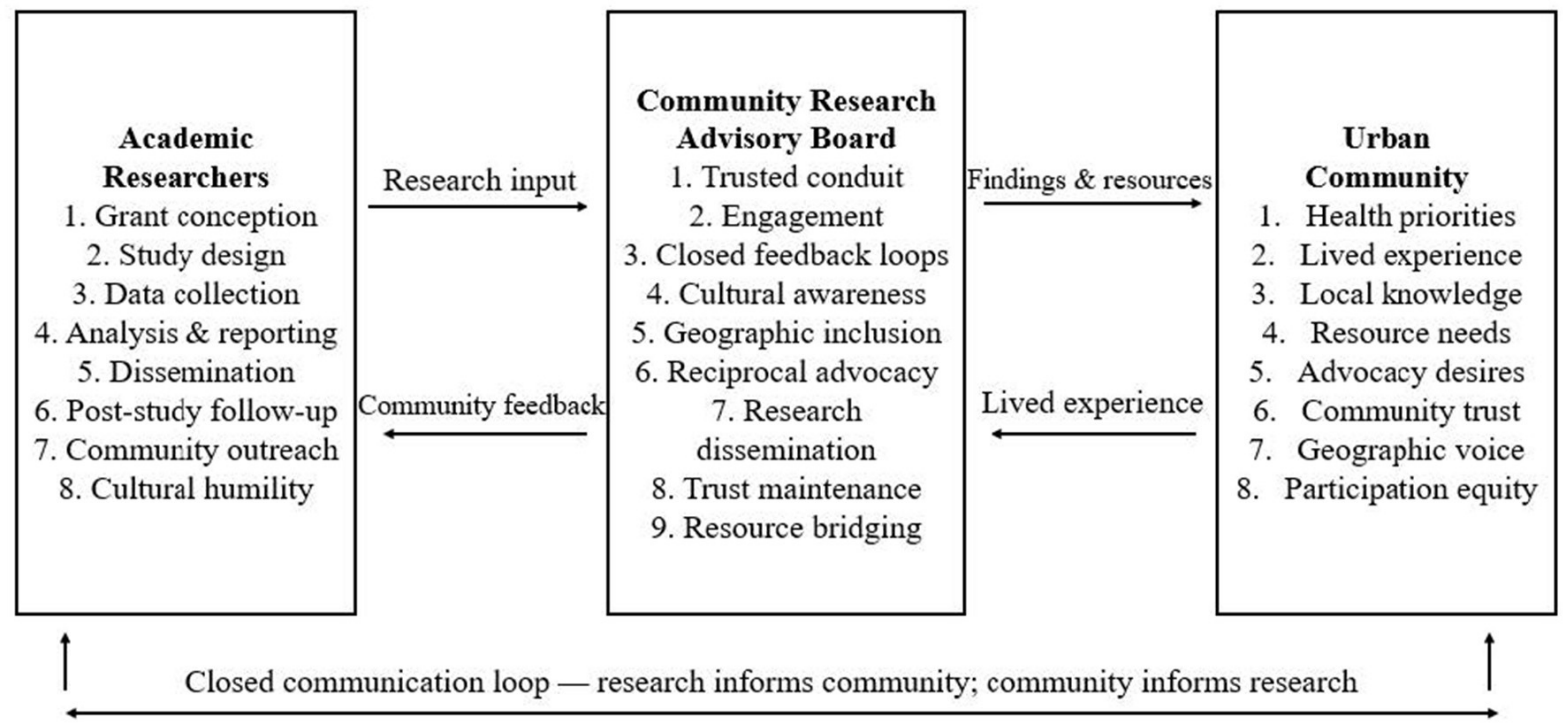
Conceptual model of effective community research partnerships through CRAB engagement.

Based on the findings of the present study, as well as previous CAB and CRAB literature, Figure 1 shows a conceptual model of effective community research partnership through CRAB engagement, particularly illustrating the CRAB as a connector between the academic researchers and the urban communities of which they are interested in positively affecting.

## Discussion

The themes highlight real-world experiences and evident spaces for growth while collectively demonstrating a common expectation for urban communities to be involved in research conducted alongside them (e.g., community-based and community participatory research). The themes developed illustrate urban communities' expectations for research-based partnerships, including meaningful partnerships that go beyond standard recruitment efforts. Research partnerships that provide a sense of meaningful impact allow urban communities to provide timely feedback, tangible advocacy, and resources while respecting local knowledge and geography (27–30). The themes describe a critical need for equitable, sustained, and impact-focused partnerships.

The present study's findings echo previous findings focused on CAB engagement (28). The present study's CRAB seemed to function similarly to the RRBs discussed in Wilczek et al. (5). Although not directly related to the university's institutional review board, the present study's CRAB provided assistance across various researchers and research projects conducted within the center. Another similarity to Wilczek et al. was that the present study's CRAB members specifically mentioned that they desired to be a part of all phases of the research process, not just for recruitment at the beginning or dissemination at the end. They expressed wanting to be included in the research decisions about the involvement of their community members (Theme 1—Urban *Communities Want Feedback and Sustained Engagement*). Although both Chassler et al. (7) and the present study collected qualitative data from the advisory boards, the present study was distinct from Chassler and colleagues examined CAB members' perceptions and personal benefits of participation. The present study not only allowed the CRAB members to speak from their experiences, but also on behalf of their community constituents, and the advocacy for their communities drove the development of the themes. The present study addresses one of Chassler's limitations, which included the prompts not addressing disadvantages. CRAB members in the present study were honest about their negative perceptions of CRAB participation, noted in theme 4, *Consequences and Benefits for the Community Connector*, where various CRAB members reported positive experiences, some reported negative experiences with room for improvement, and some were situated in the middle regarding how they perceived the benefits.

Connors and colleagues (11) emphasized the value of community expertise. This was a central feature that rang true throughout the present study's themes, particularly themes 1 and 2, Urban *Communities Want Feedback and Sustained Engagement* and Urban *Communities Feeling Ignored or Used by Researchers*. Halladay and colleagues (12) highlighted a similar recommendation of early member involvement, clear roles, and sustained partnership engagement for CABs. Additionally, Connors and colleagues found success in ensuring that CAB members' feedback was incorporated in research studies via real-time meeting notes and anonymous feedback evaluations. This is similar to our findings that emphasized the incorporation of board feedback in the promotion of satisfied involvement (Theme 1: Urban *Communities Want Feedback and Sustained Engagement*). These changes addressed their previous low CAB member attendance, which may have been due to a lack of clear communication and involvement. This suggests that while CABs and CRABs play a critical role in health research, their effectiveness is often compromised by structural and operational challenges. This may echo Stewart et al. (4), who found that some CAB participants struggled to contribute meaningfully.

The present study adds to the existing literature on CAB engagement by specifying particular concerns about working with urban communities and applying the findings specifically to urban academic-community partnerships. Through the use of reflexive thematic analysis, we are able to develop more personalized findings on behalf of the board members, rather than process and performance data via evaluation surveys. To this end, our study uniquely highlighted localized geographic neglect in areas that are heavily populated by persons of color. Additionally, the present study explored the benefits and challenges of using a community connector (Theme 4: *Consequences and Benefits for the Community Connector*). We can observe a “domino effect” of sorts and an evolving community expectation for inclusive involvement in community research with university researchers. Our findings describe how neglect or exploitation of one partnership may negatively impact multiple community relationships and, thereby, harm institutional credibility. This demonstrates that partnerships are not isolated to a singular, independent research partnership; rather, the community connector is a liaison to broader organizations that represent both the university researchers (for the community) *and* the community organizations (for the university researchers). As a mediator between the two, the CRAB was specifically concerned that the lack of engagement on the part of the research team, after they've reaped the benefits of the communities, may damage the CRAB members' trust and reputation. Our findings suggest that their involvement should extend beyond helping the research and ensure that the research, in turn, helps them (e.g., Theme 1—*Urban communities want feedback and sustained engagement* and Theme 3—*Urban communities want advocacy and resources, not just an identification of the problem*).

### Implications for practice

Taken together, we will establish partnerships with CABs and CRABs that prioritize genuine reciprocity and mutual accountability to ensure community engagement. Their involvement needs to extend throughout the project lifecycle (e.g., grant conception, planning, analysis, dissemination, post-study follow-up) and ensure that the research is disseminated back to them. Researchers might also strengthen their relationships with sensitivity to geographic blind spots (e.g., service gaps) that often involve their community constituents. Documenting input could maximize the board members' trust (30).

Based on the findings, checking in is particularly important during “down times” when researchers are not in direct need of community members for participation or dissemination, for example. Checking in with community organizations by providing research status updates or clarifying the study's expectations of what's next is an excellent way to sustain engagement. Providing health guideline updates, health literacy materials, or known services and resources for the community, for example, helps sustain relationships and builds trust through mutually beneficial outcomes for urban communities. As a Community Engagement Core, we intend to sustain engagement by establishing a bi-directional information flow between CRAB members and the researchers. This implies that there is a closed loop, meaning researchers followed up and debriefed the CRAB and community upon the conclusion of the study.

### Study limitations

Several limitations should be considered when interpreting the findings of this study. The sample was drawn from a single CRAB affiliated with one academic institution. These findings are not generalizable to CABs and CRABs. It is simply a contribution of one CRAB's perspective on their service to academic research.

- This study relied on focus group data from a small sample size (*N* = 7), which may limit the perspectives captured.
- Although two focus groups were scheduled and conducted, only one person showed up for the first focus group opportunity, which may be considered a dyadic interview more so than a traditional focus group. Nonetheless, the interaction between the CRAB and the researchers in both facilitations captured rich data and co-construction of meaning, which is a cornerstone of focus group interaction.
- Because all participants identified as women, findings may not capture male perspectives regarding community-research partnerships. It is unclear whether a male gendered perspective would influence the interactions, data reflection, or interpretation; therefore, potential gender bias should be acknowledged.
- The researchers acknowledged that their professional roles within the University of Houston HEALTH Center and prior relationships with CRAB members may influence data interpretation.
- Finally, the study reflects participants' perceptions and lived experiences, which are shaped by historical and local context. Although reflexive thematic analysis allows for researcher positionality to be acknowledged, the interpretation of themes remains influenced by the research team's perspective.

Despite these limitations, this study provides actionable insight into how urban CRAB participants may experience academic research partnerships.

## Conclusion

Community connectors have a place in research, and research has a place in the community. “No research about us, without us,” means that the community is more than a tool to assist research. Rather, communities desire to give input on the systems and science that govern their lives. Communities are beneficial stakeholders in their needs when equipped with knowledge, skills, and support. To fully realize the potential of CABs and CRABs, there is a need for concerted efforts to address challenges, including regulatory oversight, improved training, clear guidelines on their roles and responsibilities, and the utilization of CAB/CRAB recommendations.

## Data Availability

The raw data supporting the conclusions of this article will be made available by the authors, without undue reservation.
